# Virtual Screening on Marine Natural Products for Discovering TMPRSS2 Inhibitors

**DOI:** 10.3389/fchem.2021.722633

**Published:** 2021-10-12

**Authors:** Mehdi Mahmudpour, Iraj Nabipour, Mohsen Keshavarz, Maryam Farrokhnia

**Affiliations:** ^1^ The Persian Gulf Marine Biotechnology Research Center, The Persian Gulf Biomedical Sciences Research Institute, Bushehr University of Medical Sciences, Bushehr, Iran; ^2^ The Persian Gulf Tropical Medicine Research Center, The Persian Gulf Biomedical Sciences Research Institute, Bushehr University of Medical Sciences, Bushehr, Iran

**Keywords:** SARS-CoV-2, COVID-19, tmprss2, molecular modeling, molecular docking

## Abstract

Although SARS-CoV-2 entry to cells strictly depends on angiotensin-converting enzyme 2 (ACE2), the virus also needs transmembrane serine protease 2 (TMPRSS2) for its spike protein priming. It has been shown that the entrance of SARS-CoV-2 through ACE2 can be blocked by cellular TMPRSS2 blockers. The main aim of this study was to find potential inhibitor(s) of TMPRSS2 through virtual screening against a homology model of TMPRSS2 using the library of marine natural products (MNPs). The homology modeling technique for generating a three-dimensional structure of TMPRSS2 was applied. Molecular docking, MM-GBSA and absorption, distribution, metabolism, excretion (ADME) evaluations were performed to investigate the inhibitory activity of marine natural products (MNPs) against TMPRSS2 and their pharmacokinetic properties. Camostat and nafamostat mesylate were used as the standard inhibitory molecules. Seven MNPs were able to inhibit TMPRSS2 better than the standard compounds. MNP 10 with CAS number 107503-09-3, called Watasenia β-D- Preluciferyl glucopyrasoiuronic acid, was found to be the best inhibitor of TMPRSS2 with acceptable pharmacokinetic properties. Herein, for the first time, a new marine natural product was introduced with potent inhibitory effects against TMPRSS2. MNP 10 exhibited favorable drug-like pharmacokinetic properties and it promises a novel TMPRSS2 blocker to combat SARS-CoV-2.

## Highlights

1) Marine natural products (MNPs) are a valuable source for anti-SARS-CoV-2 drugs.
2) MNP 10 is a potent TMPRSS2 inhibitor to combat SARS-CoV-2.
3) MNP 10 has favorable drug-like pharmacokinetic characteristics.

## Introduction

The devastating pandemic caused by SARS-CoV-2 (Astuti, 2020; Guan et al., 2020; Wang et al., 2020; Zhu et al., 2020) that first broke out in Wuhan, China in late 2019, has become the most important global health and socioeconomic issue. Although there is a worldwide effort to develop an effective vaccine against SARS-CoV-2 using both established and new vaccine production technologies (WHO, 2021), no one can yet claim what kind of therapy can be absolutely efficient for the treatment of or protection against COVID-19. Based on the involved pathophysiologic pathways, different kinds of therapeutic modalities have been conducted in numerous clinical trials with conflicting results.

SARS-CoV-2 is a spherical shaped virus with a diameter of about 60–140 nm with some pleomorphism belonging to Coronaviride family. The enveloped virus has distinctive spikes (Zhu et al., 2020). Its genome is around 29.8 kilobase with a single-stranded positive-sense RNA (Lu et al., 2020a; Chan et al., 2020; Zhou et al., 2020) encoding 12 putative structural and non-structural proteins; of which spike(S), envelope (E), membrane (M) and nuclecapsid (N) proteins are structural. The S protein complex has two subunits, the S1 subunit contains a single peptide, a receptor-binding domain (RBD) which mediates attachment of virion to host cell surface receptors, and an N-terminal domain (NTD). The S2 subunit mediates fusion between the viral and host cellular membranes which facilitates virus genome entry into the host cell (Gui et al., 2017; Kirchdoerfer et al., 2018; Song et al., 2018; Wan et al., 2020).

Although it has been discovered that the SARS-CoV-2 entry to cells strictly depends on Angiotensin-Converting Enzyme 2 (ACE2) (Li et al., 2003; Li and De Clercq, 2020), it has been shown in several studies that SARS-CoV-2 also needs transmembrane serine protease 2 (TMPRSS2) for S protein priming (Hoffmann et al., 2020a; Hoffmann et al., 2020b; Stopsack et al., 2020). Hoffmann et al. showed that the entrance of SARS-CoV-2 through ACE2 can be blocked using cellular TMPRSS2 blockers. The SARS-CoV-2 spike protein contains several arginine residues with a high cleavability action at the communication point in the S1/S2 cleavage site. It has been suggested that this zoonotic-origin cleavage site sequence is required for SARS-CoV-2 entrance into human cells. These findings are consistent with previous observations from several clinically relevant viruses such as MERS, other Coronaviruses, and Influenza A virus (Kim et al., 2006; Matsuyama et al., 2010; Glowacka et al., 2011; Shulla et al., 2011; Kawase et al., 2012; Gierer et al., 2013; Zhou et al., 2015; Shen et al., 2017; Iwata-Yoshikawa et al., 2019; Kleine-Weber et al., 2019). Moreover, Heurich et al. showed that TMPRSS2 and other potentially related proteases cleave the ACE2 and SARS-S protein leading to the SARS-CoV entry and fusion of the virus S protein with the host cell membrane, respectively (Heurich et al., 2014). Therefore blocking S protein priming by specific serine protease might have the potential to control SARS-CoV-2 infection. There are some therapeutic agents like bromehexine (Lucas et al., 2014), camostat mesylate (Shirato et al., 2013), and nafamostat mesylate (Yamamoto et al., 2016) that have been elucidated to be a good inhibitor of TMPRSS2.

Regarding TMPRSS2 inhibitors as potent candidates for anti SARS-CoV-2 infection, it may be promising to investigate natural resources to discover novel components with anti-TMPRSS2 activities. The extreme and unusual environment of the ocean has extraordinary organisms with astonishing properties, which can reveal new horizons for treatment in modern medicine including marine-derived secondary metabolites with evident anti-inflammatory, antitumor, antimicrobial, antiviral, antimalarial, and antioxidant activities (Molinski et al., 2009; Gogineni et al., 2015; Blunt et al., 2018; Riccio et al., 2020; Yi et al., 2020; Carroll et al., 2021). Based on the extraordinary self-defense capacities of marine organisms and the occurrence in them of some deadly viral infections, these organisms might be regarded as a source of novel antiviral agents, which may be able to combat a SARS-CoV-2 infection. Hence, marine-derived natural compounds should be considered in our efforts to overcome the challenges of COVID-19 treatments.

A promising method to investigate viral entry and proliferation is to apply a computer-aided active site directed inhibition study. To the best of our knowledge, there are no studies that have screened the marine natural products (MNPs) libraries specifically to find a blocker of TMPRSS2. However, several studies have screened the libraries of other natural products, particularly plant-derived compounds, to discover the potential inhibitors of TMPRSS2 (Chikhale et al., 2020; Da Silva Antonio et al., 2020; Idris et al., 2020; Rahman et al., 2020; Singh et al., 2020; Vivek-Ananth et al., 2020; Hu et al., 2021).

This study aimed to find potential inhibitor(s) of TMPRSS2 through virtual screening against a homology model of TMPRSS2 using the library of marine natural products (MNPs).

## Materials and Methods

### Homology Modeling

The essential step in the study of the structural and functional aspects of any protein is to have its suitable crystal structure. Unfortunately, the three-dimensional (3D) structure of TMPRSS2 had not been found at the time of the current study. Hence, in this case, the only option was to generate a 3D coordinate of TMPRSS2 by comparative prediction approach. Herein, the online server SWISS-MODEL (Guex et al., 2009; Bertoni et al., 2017; Waterhouse et al., 2018) (https://swissmodel.expasy.org/) was used to build the 3D structure of TMPRSS2. The amino acid sequence of human transmembrane protease serine 2, from the Universal Protein Resource “UniProtKB” (accession no: O15393) isoform-2,492 amino acids long (https://www.uniprot.org/uniprot/O15393), was selected for homology modeling that was performed by a template-based method. Then, the RAMPAGE server was used to validate the 3D modeled structure (http://mordred.bioc.cam.ac.uk/∼%7B%7Drapper/rampage.php).

### Pharmacophore-Based Virtual Screening

The pharmacophore model was created using the Pharmit server (http://pharmit.csb.pitt.edu/). First, pharmacophore features were automatically extracted from the co-crystalized inhibitor of Serine protease Hepsin (PDB: 5CE1.A), and camostat mesylate was also used as another ligand for the pharmacophore modeling to obtain a more realistic model for homology structure of TMPRSS2 as input receptor. Hence, the pharmacophore model used for TMPRSS2 virtual screening was generated based on the predicted binding interactions of TMPRSS2 with these two inhibitors. The Pharmit parameters for 3D-pharmacophore research were changed according to these pharmacophore parameters. Then the MNP library, which contains 164,952 conformers from 14,064 molecules, was searched on this model. The hit compounds with an RMSD ≥4 Å and minimized affinity ≥ -6 were discarded. The remaining poses were minimized using functions of Pharmit. Finally, the pharmacophore-based minimized entries were further docked with the target protein to identify the lead compounds with the best docking scores.

### Active Site Identification and Preparation of TMPRSS2 for Docking

The conserved domain (CD) search was done on the Fasta sequence of the homology model of TMPRSS2 using the NCBI’s conserved domain database (CDD/SPARCLE: https://www.ncbi.nlm.nih.gov/cdd/) (Lu et al., 2020b). Then, it was analyzed and its cleavage, active, and substrate binding site residues were predicted. The COACH-D server (https://yanglab.nankai.edu.cn/COACH-D/) (Yang et al., 2013; Wu et al., 2018) was also applied to predict TMPRSS2 putative ligand-binding sites. Then, in a model-template active site comparative study, the active and substrate binding residues of the (5CE1.A) template were similarly obtained.

Finally, the homology structure of TMPRSS2 was imported into Maestro Protein Wizard and sitemap analysis was done by SiteMap (Halgren, 2007; Halgren, 2009; Schrödinger, 2015b).

This software was used with default settings, in which the top five possible binding sites by a minimum of 15 points were identified while cropping site maps set at 4 A from the nearest site point. SiteMap used a more restrictive definition of hydrophobicity by standard grid. A SiteScore value above 0.80 is indicative of high druggability and promising drug-binding sites and is used in conjunction with Dscores, which serve as a measure of hydrophobicity. Dscore or druggability score penalizes increasing hydrophilicity and is thus used as a druggability measure for a pocket. In general, Dscore <0.83 is considered as “undruggable,” 0.83–0.98 as “difficult to drug” and >0.98 as “druggable” (Halgren, 2007; Halgren, 2009; Vidler et al., 2012).

### Molecular Docking

#### Ligand Preparation

One conformation was generated per compound which was followed by geometry optimization with PM3 (Stewart, 1991), a semi-empirical method using Hyperchem release 7 for windows (HyperCube Inc., 2002). The geometry-optimized structures were retrieved in MOL type for further analysis with the LigPrep application which has been implemented in the Schrödinger 2015-2 suite of software (Schrödinger, 2015a). The ionization state was specified at pH = 7.00±2.0 using Epik (Epik, 2015) based on Hammett and Taft methodologies (Sastry et al., 2013). The desalt option was the same as the program default. All 32 possible conformations were produced for each compound at pH 7.00 in the OPLS3 (Harder et al., 2016). The obtained ligands were then used in the docking calculations.

#### Generation of the Grid

As adequate generation of the grid is a key step in the prediction of a ligand binding to a receptor, the 3D boundary for ligand binding was produced by Glide, version 10.2 of Mastero, Schrödinger (Glide, 2015). First, the protein preparation wizard was used with the following settings: 1) The original hydrogens removal and subsequent addition of hydrogens. 2) The atomic charges and bond orders were assigned. 3) The N and C termini were capped. 4) The disulfide bonds were generated between sulfur atoms (within 3.2 Å). 5) Epik was applied to generate possible protonation states at neutral pH. 6) The H-bonds were assigned, optimized by PROPKA (Olsson et al., 2011; Søndergaard et al., 2011) at pH 7.0, and then the structure was minimized with the OPLS3 force field. Glide was used to generate the grid on the catalytic domain of the receptor. The grid box size was set to 32*32*32 Å.

#### Molecular Interaction and Docking Studies

After grid generation, ligand docking was done according to the protocols in Glide version 10.2. The homology model of TMPRSS2 was used as the receptor, and the different internally produced conformations by the software were passed across some filters such as Euler angles, grid-based force field evaluation, and energy minimization by Monte Carlo. Finally, docking score is an important parameter for evaluating the conformations, and in this study, the output of standard precision (SP) docking was put forward in extra precision (XP) docking. The docked compounds were ranked based on their docking scores.

### Pose Rescoring With Molecular Mechanics Generalized Born Surface Area

The binding energies of all docking poses were calculated using the molecular mechanics generalized Born surface area (MM-GBSA) approach implemented in the Prime program in the Schrödinger software suite (Prime, 2015). This approach employs a single minimized protein-ligand structure, and so is used as an efficient approach to rapidly refine and rescore docking results. A variable dielectric solvent model VSGB 2.0 (Li et al., 2011) was used, this solvent model contains several empirical corrections for modeling the directionality of hydrogen bond and *π*-stacking interactions. MM-GBSA has been shown to give good binding free energies for a wide range of protein-ligand complexes (Mulakala and Viswanadhan, 2013). It is also widely used to evaluate docking poses, to determine the stability of ligand-target complex for predicting binding affinity in drug design (Genheden and Ryde, 2011; Wang et al., 2019).

### Absorption, Distribution, Metabolism, and Excretion and Drug-likeness Analysis

It is known that nearly 40% of drug candidates fail in clinical trials because of poor absorption, distribution, metabolism, and excretion (ADME). Hence, it is very crucial to recognize these problematic candidates at an early stage to avoid wasted time and resources. Accurate ADME prediction is based on full 3D molecular structures. Qikprop offers a set of several predictors including central nervous system (CNS) penetration, predicted apparent Caco-2 cell permeability across the gut-blood barrier in nm/sec (QPPCaco), apparent MDCK cell permeability (QPPMDCK), human oral absorption, Lipinski’s rule of five, and predicted maximum transdermal transport rate (JM). Another option of Qikprop (Qikprop, 2015) is to rank compounds based on how drug-like they are. In the current study, the ADME, drug-likeness, and medicinal chemistry parameters of these 11 compounds were predicted by QikProp (Qikprop, 2015).

## Results and Discussion

### Homology Modeling and Evaluation of Model Quality

**FIGURE 1:**
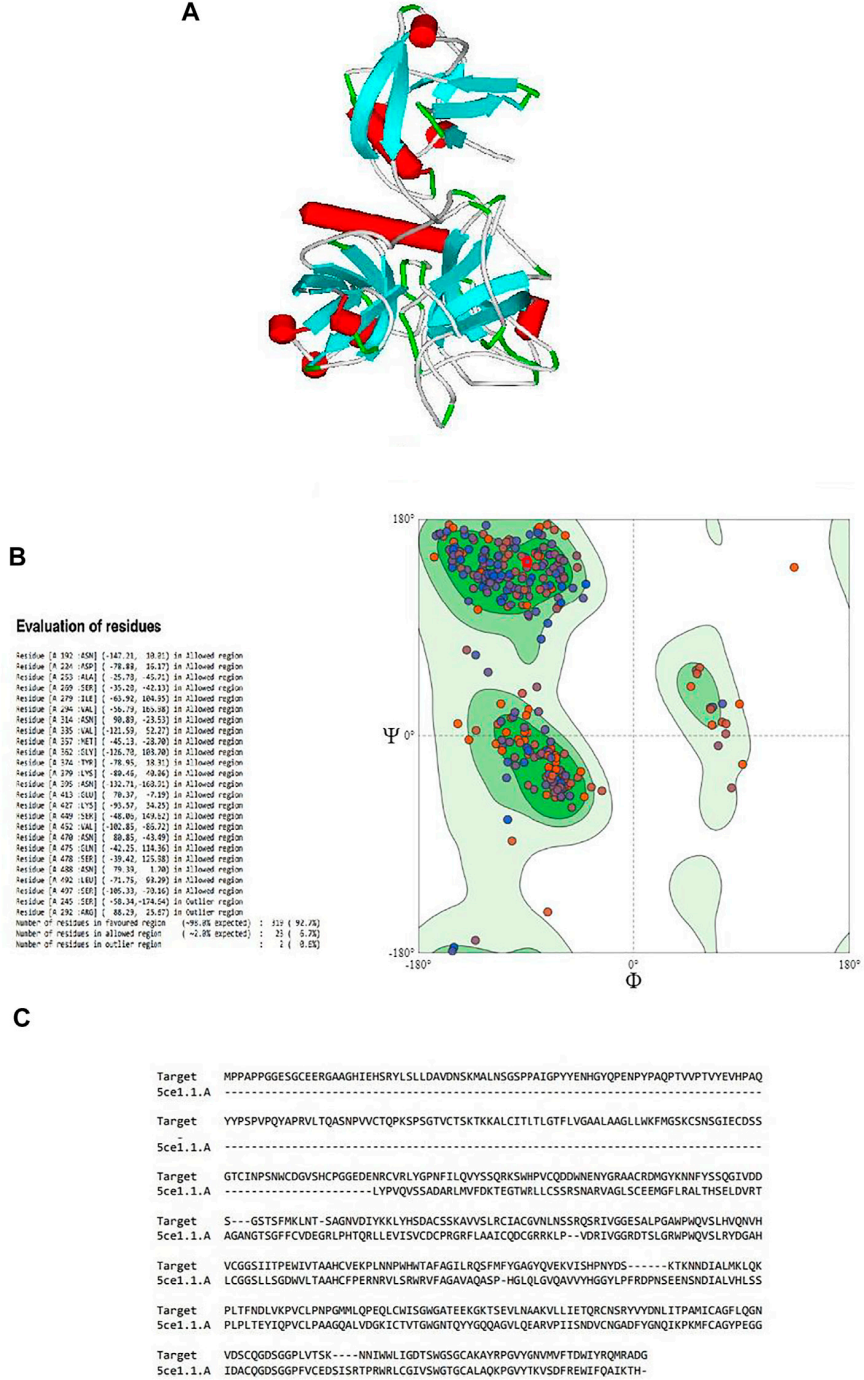
**(A)** Three-dimensional structure of the modeled serine protease transmembrane protease serine 2 (TMPRSS2), **(B)** Ramachandran plot validation of the modeled 3D structure, **(C)** alignment of the target serine protease TMPRSS2 and the template serine protease hepsin (PDB ID: 5CE1.A.).

Since the crystal structure of TMPRSS2 was unavailable at the time of this study, the 3D structure of TMPRSS2 was predicted using the online server SWISS-MODEL, as shown in Figure 1A. Its global model quality estimate (GMQE) score was 0.48, this score estimates the quality of the expected output model by a particular template. Its QMEAN Z-score was -1.47, with sequence coverage of 71%, sequence identity of 33.82%, and sequence similarity of 50% in comparison with the template (PDB ID: 5CE1.A). It has been shown that when the sequence similarity with the template is more than 30%, the obtained model can be considered reliable and suitable for further study (Xiang, 2006). Benkert et al. (2011) showed that QMEAN Z-score is an estimation of the degree of native likeness of the model, and a value close to 0 (and not lower than -4) could be an acceptable agreement criteria for the experimental structure of similar size Table 1.

**TABLE 1:** Homology modeling results and validation for predicting 3D structure of TMPRSS2.

Server/tool	Parameter	Score
Swiss-model	QMEAN z score	−1.47
	GMQE	0.48
	Seq Similarity	51%
	Coverage	71%
	Seq identity	33.82%
RAMPAGE(Ramachandran plot)	favored region	92.7%
	Allowed region	6.7%
	Outlier region	2%
MolProbity	Molprobity Score	1.89
	Ramachandran Favoured	92.20%
	Ramachandran Outliers	1.16%
	Rotamer Outliers	1.35%
	C-Beta Deviations	7%

By validating the obtained results of SWISS-MODEL and cross-checking in RAMPAGE, it was observed that there were 319 (92.7%) residues were in the favored region, 23 (6.7%) residues in the allowed region, and 2 (0.6%) residues in the outlier region. Moreover, there were no steric clashes or deviations in bond length or bond angle compared to the protein structure report (Supplementary Figure S1 and Supplementary Figure S2). These results indicated that the obtained model may have the correct geometry. The 3D arrangement of the model is shown in Figure 1B. The alignment of the template (PDB ID: 5CE1.A) and the target protein is shown in Figure 1C. The summary of obtained results is presented in Table 1.

### Catalytic Site of the Homology Model of TMPRSS2

According to a conserved domain database (CDD) search, the protein classification of TMPRSS2 was Trypsin-like serine proteases with E-value 7.39e-100, and the residues of ILE293-GLN524 were involved in the characteristic domain of this protein. Based on the CDD algorithm, six amino acid residues have been identified as especially important in the active site of TMPRSS2, and one residue for its cleavage site. The active site included residue HIS333 to SER478 where (HIS333, ASP382, and SER478) were the three important amino acids at the catalytic site whereas (ASP472, SER497, and GLY499) residues were found to be the substrate binding site (Table 2). Moreover, the COACH-D results supported those findings. According to the COACH-D best prediction results, the protein template was a serine protease of the coagulation system (PDB ID: 5JB8) with a confidence score of 0.99.

**TABLE 2:** Predicted binding site of homology model of TMPRSS2.

Predicted binding site	C-score	E-Value	Involved residues	Docking energy of representative ligand-template complex	Predicted binding residues
CDD-search	—	7.39e-100	ILE293-GLN524	—	HIS333 to SER478
					Catalytic site: HIS333, ASP382 and SER478
					Substrate binding site: ASP472, SER497and GLY499
COACH-D	0.99	—	ILE293-GLN524	−6.0	HIS333, LYS379, ASP472-GLY476, SER478, THR496-CYS502 and GLY509

The corresponding docking energy of the template for its representative ligand (with pubchem CID: 137347860) was -6.0 kcal/mol. HIS333, LYS379, ASP472-GLY476, SER478, THR496-CYS502, and GLY509 were identified as the predicted binding residues, of which the CDD search showed HIS333, ASP472, SER478, SER497, and GLY499, as the same catalytic domain residues.

**FIGURE 2:**
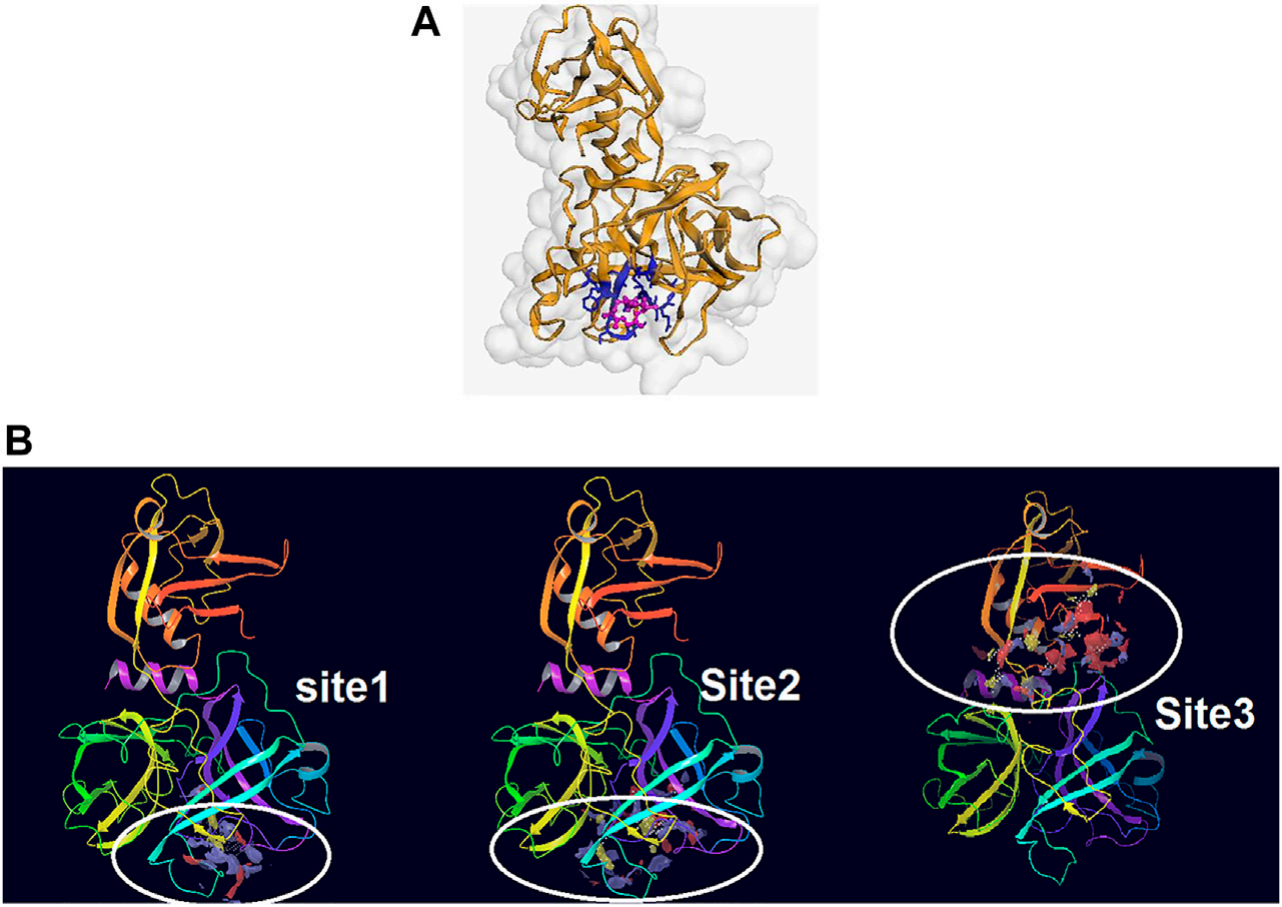
**(A)** The predicted binding site by CPACH-D server. The protein template is a serine protease of the coagulation system (PDB: 5jb8) and ligand is [(4S, 5S)-4-[[2-[[(2S)-2-amino-4-carboxybutanoyl]amino]acetyl]amino]-6-chloro-5-hydroxyhexyl]-(diaminomethylidene)azanium with pubchem CID: 137347860. **(B)** The binding sites predicted by Sitemap. Site1: Sitescore 0.972, Dscore 1.001, size 87, and volume 150. Site 2: Sitescore 0.968, Dscore 1.026, size 91, and volume 228. Site 3: Sitescore 0.994, Dscore 1.044, size 108, and volume 331.

From the five binding sites which were calculated by sitemap, two sites had a Dscore above 1, and their locations were completely in the agreement with the CDD search and COACH-D results (Site 1: Sitescore 0.972, Dscore 1.001, size 87, and volume 150. Site 2: Sitescore 0.968, Dscore 1.026, size 91, and volume 228). The Dscore of site 3 was 1.044 with Sitescore 0.994 but its position is not in agreement with the catalytic site of Trypsin-like serine proteases active domain which is located in the base of its S1 pocket where it contains Asp472, and therefore is predicted to cleave after lysine or arginine residues (Hedstrom, 2002; Wilson et al., 2005; Blay and Pei, 2019). The predicted binding sites by all applied approaches are in agreement with the theoretical study by Idris et al. (2020). Two other predicted binding sites had a Dscore lower than 0.8, which indicates that they are not druggable. Hence, domains 1 and 2 were chosen for the docking study. Figures 2A,B illustrate the predicted binding site of TMPRSS2 from COACH-D and SiteMap analysis.

### Pharmacophore Model

**FIGURE 3:**
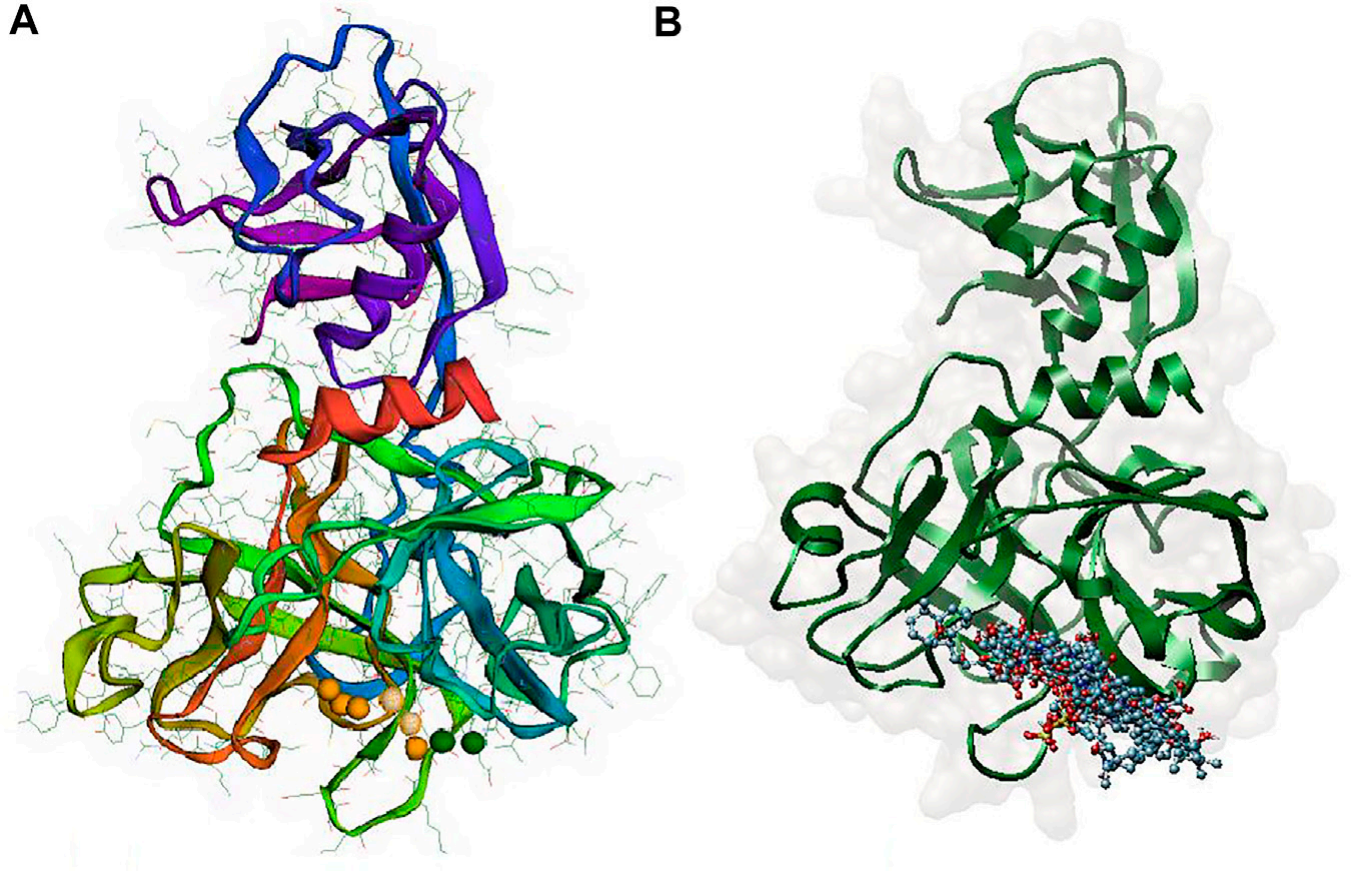
**(A)** Pharmacophore model generated by the Pharmit server, Two amide nitrogen atoms to represent hydrogen bond donors (DON) (green sphere), four negatively charged oxygen atoms (as in a carboxyl group) to represent a hydrogen bond acceptor (ACC) (orange sphere), and the two isopropyl group to represent a hydrophobic center (HYD) (yellow sphere) **(B)** superposition of all 11 aligned lead MNPs according to pharmacophore model.

Since a pharmacophore states the crucial features of interactions, such as the spatial arrangement of each interaction in the close contact of ligand and the target, its accurate setup is very important in binding site pharmacophore modeling. In this study, the 3D structure of TMPRSS2 homology was used to set pharmacophore using the Pharmit server; this server provides both pharmacophore and molecular shape search options and the results are ranked by the energy. The generated pharmacophore features were selected according to the co-crystal inhibitor of the template and camostat mesylate. In this modeling, the binding-site derived pharmacophore models include three subgroups of ligand binding sites: i) two amide nitrogen atoms were added to represent hydrogen bond donors (DON), ii) four negatively charged oxygen atoms (as in a carboxyl group) were added to represent a hydrogen bond acceptor (ACC), and iii) the two isopropyl group were added to represent a hydrophobic center (HYD) (Figure 3A). According to the generated pharmacophore model, a vast library of MNP (14,064 molecules, 164,952 conformers) was filtered. A total of 25,000 hits that met the criteria were minimized, resulting in 114 conformers. A total of 11 structures were retained by using one conformer for each molecule, with an RMSD lower than 4 Å and a binding score lower than -6 (Figure 3B and Table 3).

**TABLE 3:** CAS number, 2D and 3D presentations of investigated marine natural products.

#	CAS#	2D structure	3D structure
1	26605-16-3	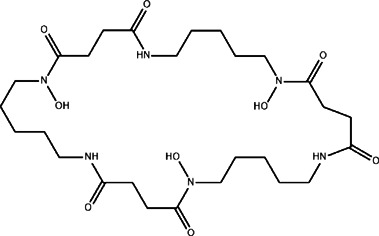	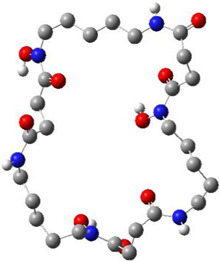
2	125127-57-3	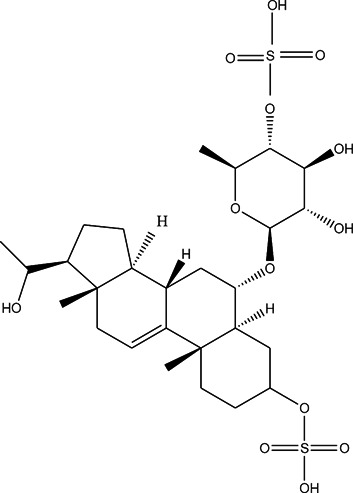	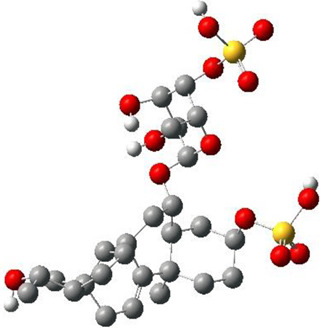
3	174286-21-6	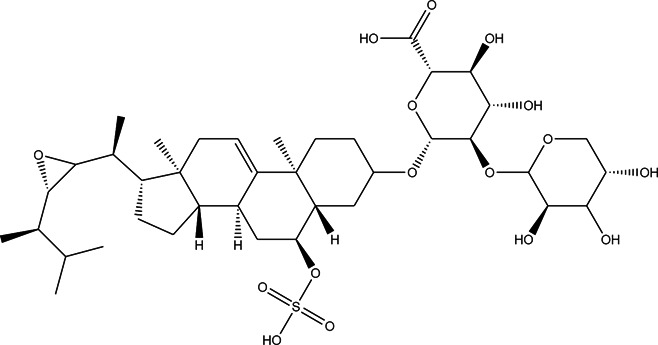	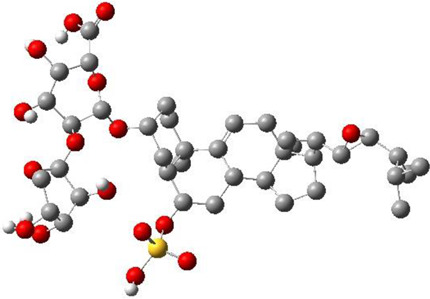
4	454470-88-3	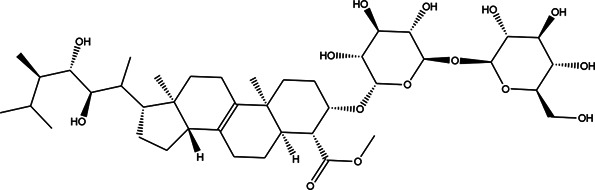	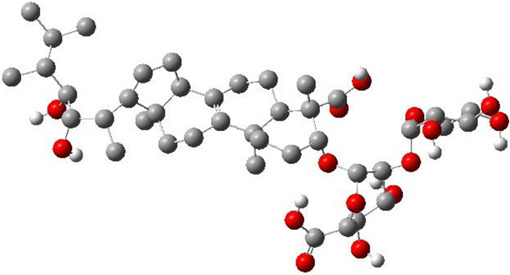
5	143572-73-0	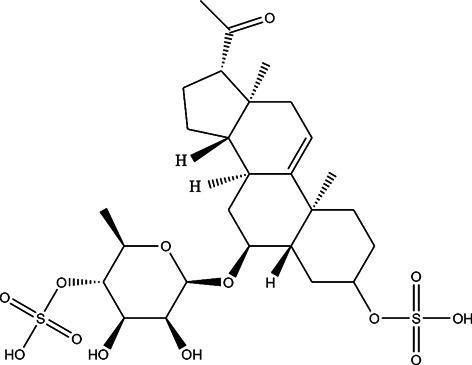	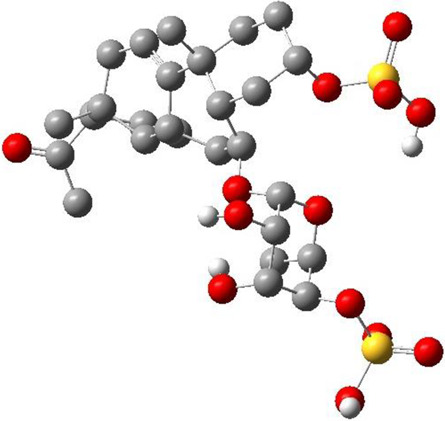
6	170894-36-7	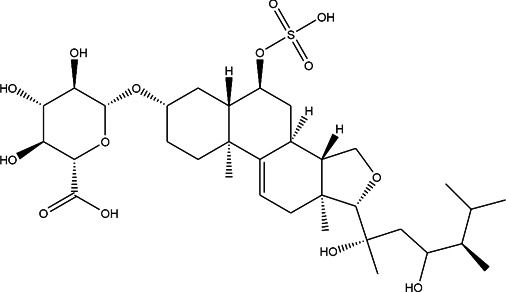	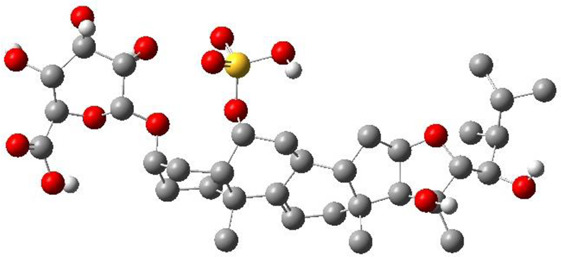
7	81720-10-7	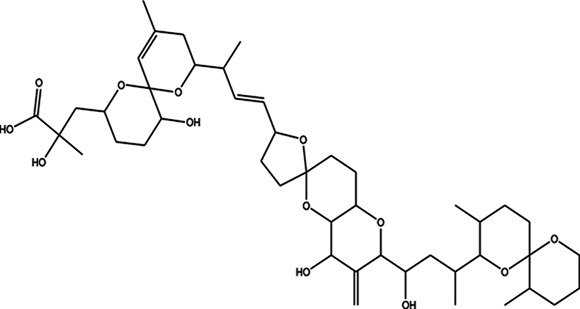	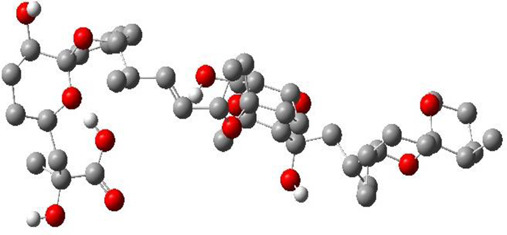
8	6184-17-4	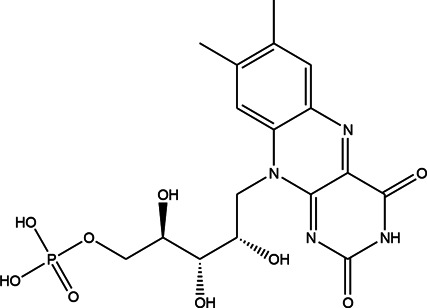	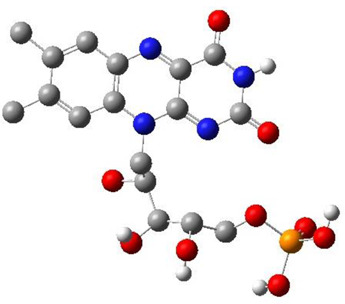
9	174286-17-0	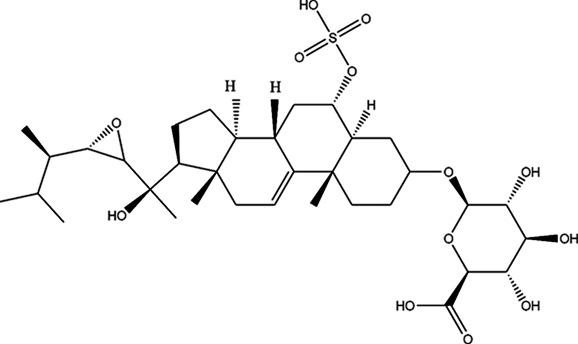	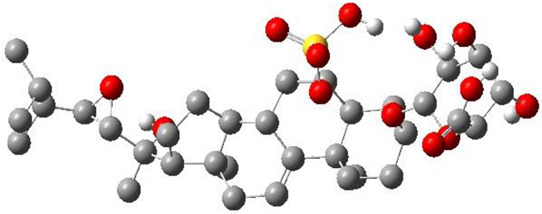
10	107503-09-3	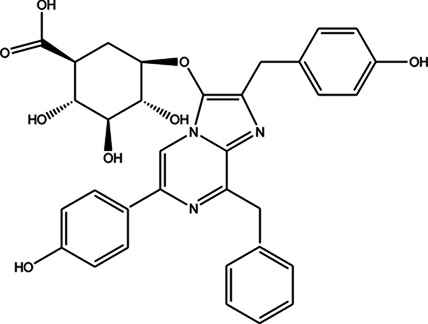	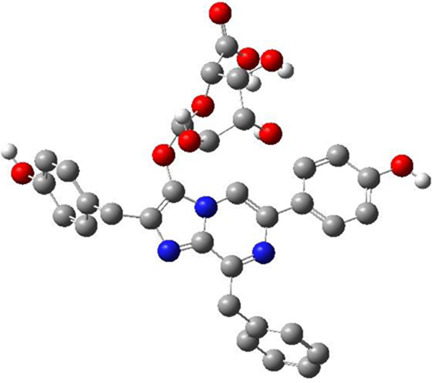
11	59985-26-1	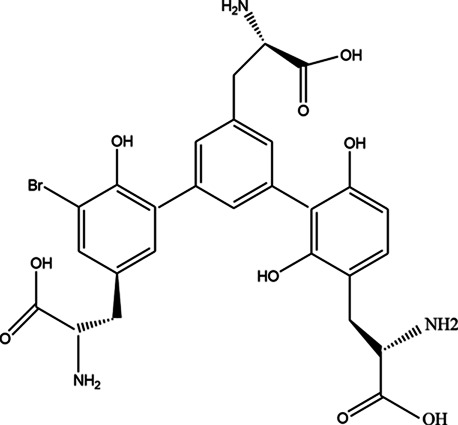	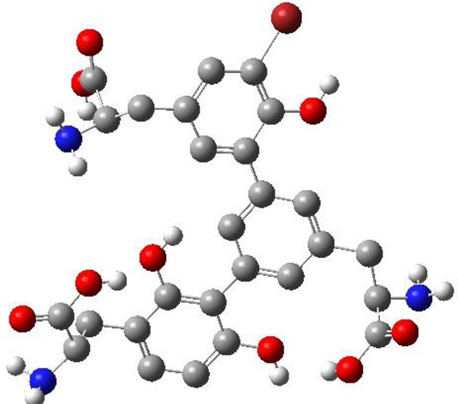

### Molecular Docking and Molecular Mechanics Generalized Born Surface Area Studies

**SCHEME 1:**
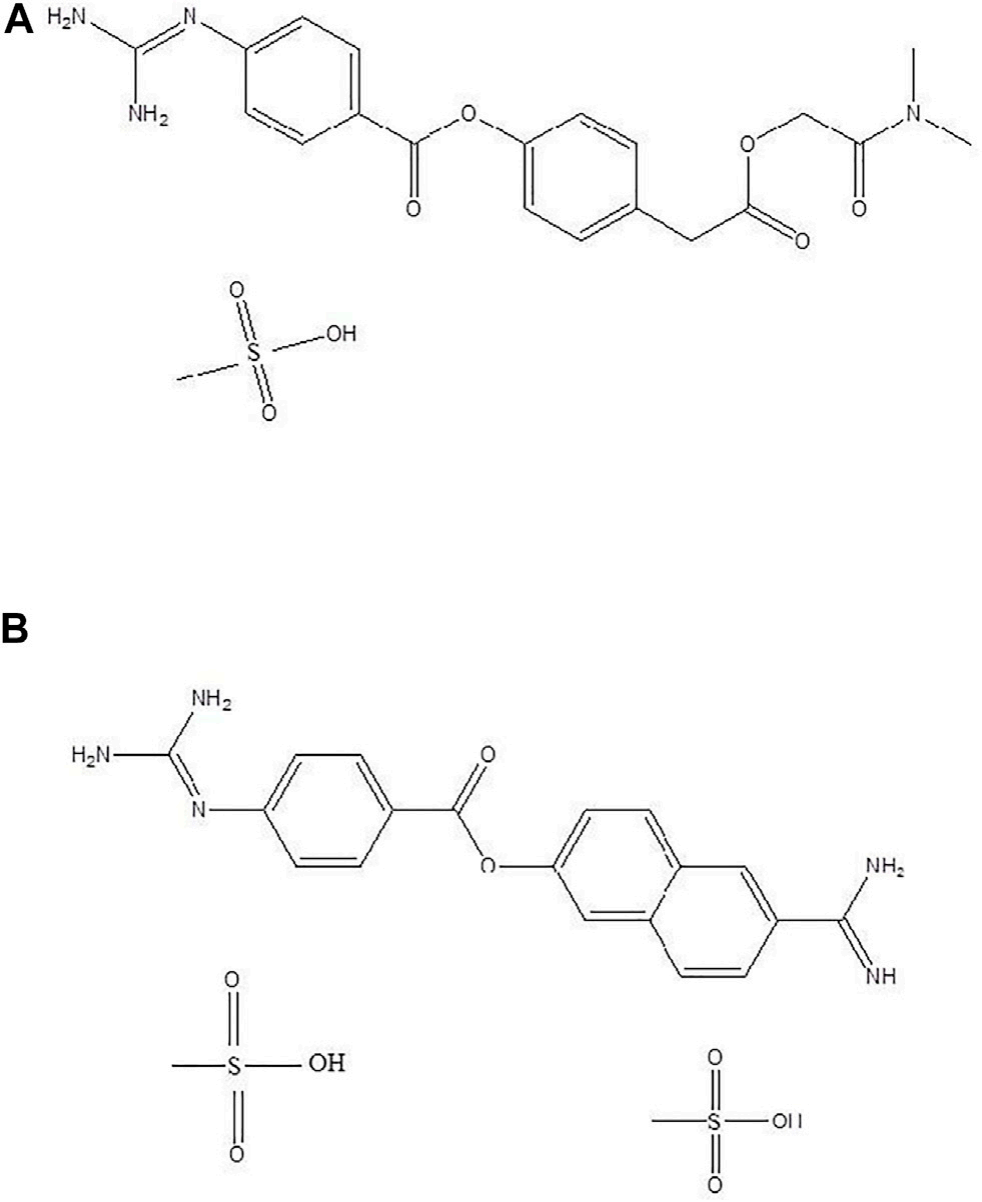
Standard inhibitors **(A)** camostat mesylate and **(B)** nafamostat mesylate.

The top 11 selected MNPs of the 114 structures which are shown in Table 3 were separately docked into the catalytic site of TMPRSS2. The results of docking of these structures, as well as camostat and nafamostat mesylate, as two standard inhibitors of TMPRSS2 (Scheme 1), are presented in Table 4.

**TABLE 4:** Glide Docking score, Glide energy, Glide emodel, and estimated free energy of binding for the best poses of investigated compounds in kcal/mol.

Ligand	Docking score	Glide energy	Glide emodel	$\Delta G_{\mathrm{bind}}$
1	−3.99	−40.89	−49.54	−36.06
2	−5.82	−47.34	−62.26	−64.33
3	−7.04	−51.70	−72.78	−73.48
4	−7.10	−47.50	−67.96	−67.30
5	−3.68	−36.62	−48.24	−54.64
6	−7.09	−42.77	−54.31	−62.99
7	−7.02	−48.18	−37.76	−53.89
8	−6.51	−47.50	−55.17	−76.00
9	−5.42	−45.15	−59.48	−67.82
10	−8.16	−59.21	−71.34	−76.00
11	−7.12	−49.37	−63.35	−67.68
Camostat	−4.52	−42.47	−53.19	−57.91
Nafamostat	−3.73	−38.27	−45.75	−47.49

The molecular docking analysis revealed that all the studied compounds had comparable or lower docking scores than those of the standard inhibitors. The highest docking Glide score is −8.16 in compound 10, whereas these scores were −4.52 and −3.73 in camostat and nafamostat mesylate respectively. Also, the docking scores in compounds 3, 4, 6, 7, and 11 are lower than −7. Thus, these MNPs could be considered as the most potent inhibitors for TMPRSS2. Moreover, the highest Glide energy value was −59.21 for compound 10, and its Glide emodel value is −71.34, which are almost the highest values. The Glide energies were −42.47 and −38.27 for camostat mesylate and nafamostat mesylate respectively, and Glide emodel values were -53.19 and −45.75 respectively. Therefore, from the observed theoretical superiority of these 11 MNPs compared to the standard inhibitors (camostat and nafamostat mesylate), these compounds may be encouraging for further studies. Interestingly, among these selected compounds, compound 10 had the most promising results. Compound 10 (CAS number 107503-09-3; Watasenia Preluciferyl β-d-glucopyrasoiuronic acid) is a bioluminescent substance that was derived from the liver of myctophina fish*, Diaphus elucens* (Inoue et al., 1987; Blunt and Munro, 2007).

**FIGURE 4:**
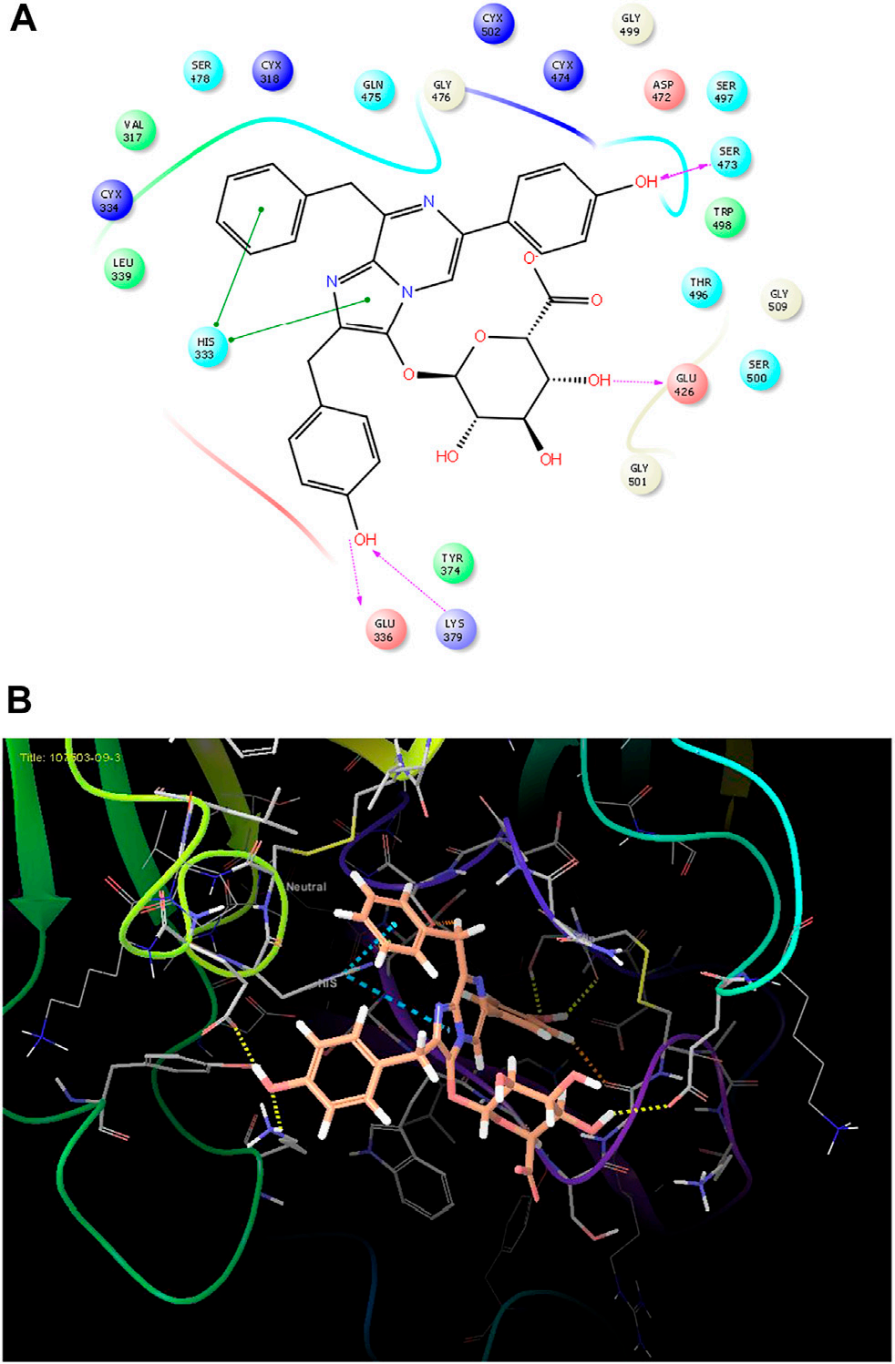
**(A)** The best pose of docking (2D) of ligand 10 at the predicted catalytic domain of TMPRSS2. **(B)** The 3D presentation of the best docking pose of ligand 10.

As it is presented in Table 5, the 2D template of best poses demonstrates the types of contacts formed between the ligands and target with cutoff 4.00 A. Remarkably, close contact/interactions within the catalytic domain were detected for all MNPs, however, all the important residues of catalytic domains significantly contributed in interactions with compound 10, such as HIS333 which was involved in *π*-*π* stacking interaction, and SER497 and GLY499 which were the important residues of substrate binding, in the close vicinity of phenol and iduronic acid moieties (Figure 4A). In addition, SER473, GLU426, LYS379, and GLU336 participated in intermolecular H-bond interaction with the active domain (Figure 4B). Taken together, compounds 3, 4, 10, and 11 had stronger interactions with TMPRSS2 than the standard ligands based on both their Glide and XP-pose emodel energies and were significantly involved in active domain contacts/interactions. On the other hand, the energy values of compounds 2 and 9 were also considerable and close to the references. Molecule 3, *Downeyoside I*, firstly isolated from starfish *Henricia Downyae* by Plagiano; molecule 2, *Forbeside E*, a sulfated sterol glycoside from starfish *Asterias forbesi*; molecule 4, *Ulososide E*, which has been derived from the sponge *Ulosa sp;* and molecule 9, *Downeyoside E,* sulfated steroid glycoside isolated from *Henricia Downeyae* (Blunt and Munro, 2007).

**TABLE 5:** 2D presentation of best Glide docking pose at the catalytic domain of TMPRSS2 for investigated marine natural product.

MNP	2D presentation of best docking pose	MNP	2D presentation of best docking pose
1	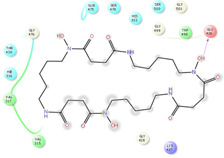	2	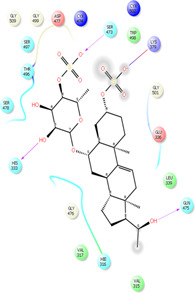
3	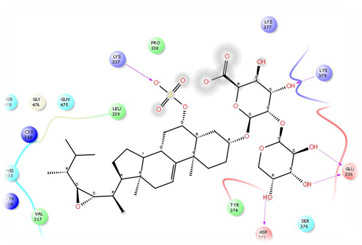	4	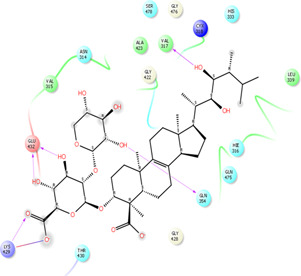
5	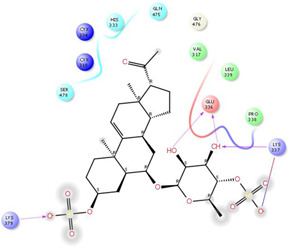	6	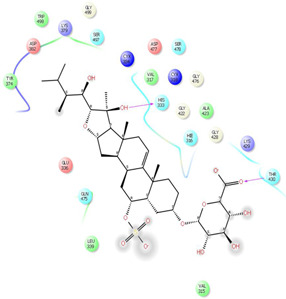
7	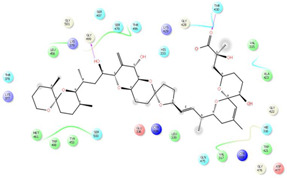	8	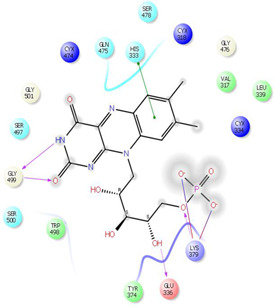
9	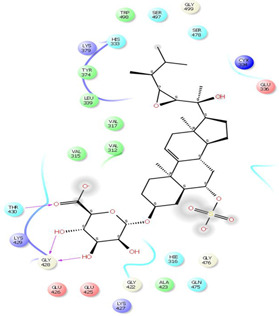	11	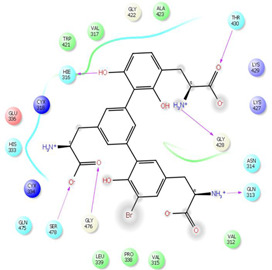
Camostate	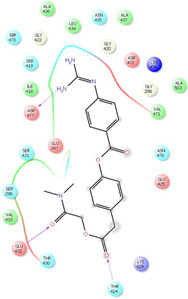	Nafamostate	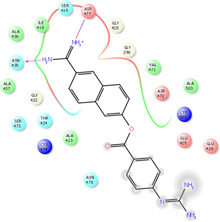

In addition, the MM-GBSA calculations which estimated the values of $\Delta G_{bind}$ are reported in Table 4. According to these results, the two standard inhibitors had values of −57.91 and −47.49 kcal/mol while eight molecules out of the 11 studied MNPs had more negative $\;\Delta G_{bind}$ than the standard inhibitors. The highest binding free energies were for compounds 8 and 10 (−76.00 kcal/mol) which is much higher than the standard inhibitors, and the values of compounds 2, 3, 4, 6, 9, and 11 were more negative than the standard inhibitors. After the rescoring of the 11 MNPs, compound 10 remained as a potent inhibitor of TMPRSS2, however other ligands might be also interesting for further studies. Although these compounds belong to various sources, starfish-derived products sound to be prominent. For example, 2, 3, and 9 have been isolated from starfish, whereas 7 has been isolated from the marine sponge Merriamum oxeato (Blunt and Munro, 2007). In this study, the marine natural products showed good measurable binding affinities for the TMPRSS2 residues. In other words, these binding affinities are indicative of the ligand’s contribution to ligand-target interactions and their sensible flexibility for this target. Based on XP Glide docking score, compounds 10 and 11 have strong interactions with the enzyme but rescoring by MM-GBSA suggests that other compounds such as 2, 3, 4, 8, and 9 are also important. Nevertheless, our proposed lead compound is compound 10, which has concurrently both high docking scores and a comparable $\;\Delta G_{bind}$ to standard inhibitors.

### Absorption, Distribution, Metabolism, and Excretion and Drug-likeness Analysis

In this study, the ADME https://www.sciencedirect.com/topics/pharmacology-toxicology-and-pharmaceutical-science/admeproperties of 11 MNPs were analyzed using the QikProp tool. This analysis represents the physicochemical properties of chemical compounds along with their biological functions. The resulting physicochemical and biological properties are molecular formula, molecular weight, volume, SASA, acceptor H-bond, donor H-bond groups, the number of ring atoms, QPlogPw (−2–6.5), the percentage of human oral absorption, and CNS effects. However, it has been suggested that Lipinski’s rule of five (Zhang and Wilkinson, 2007) is not a strict criterion for natural compounds (Lipinski, 2003) and it has been revealed that natural compounds mostly do not follow Lipinski’s rule and they tend to keep their low hydrophobicity as well as their potential of donating the intermolecular H-bonds (Ganesan, 2008).

In general, Lipinski’s rule of five (Lipinski et al., 1997) is applied for predicting the drug-likeness with the following criteria: molecular mass less than 500 Da, up to 5 hydrogen bond donors, no more than 10 hydrogen bond acceptors, and an octanol-water partition coefficient (logPo/w) no higher than 5. The rule states that a molecule or an inhibitor can be orally absorbed/active if two or more of these thresholds are not violated. However, Jorgensen’s rule of three may also be used to evaluate the bioavailability of each marine natural product by estimating its solubility, permeability, and liver first-pass metabolism through the following rules: predicted aqueous solubility (logSwat) higher than -5.7 (with S in mol/dm3), predicted apparent Caco-2 cell rate permeability (BIPcaco-2) high than 22 nm/s, and number of primary metabolites up to 7 (Di and Kerns, 2015). In addition, the predicted qualitative human oral absorption (2 = medium and 3 = high) and the predicted skin permeability (logKp values between -8.0–1.0) are considered. Finally, the ADME-compliance score drug-likeness parameter (#star) was used to evaluate the pharmacokinetic of the studied compounds, including 25 different properties within the acceptable range of 95% of the known drugs. Herein, compounds 2, 5, 9, and 10 had the fewest violations when ADME-compliance score drug-likeness parameter (#stars) was considered less than 2 (Table 6), however, the recommended value is 0–5, and compounds 6 and 8 were also considered in the acceptable range of #stars. It is concluded that these MNPs may be proper candidate drugs for TMPRSS2 inhibition.

**TABLE 6:** ADME parameters and drug similarity of investigated marine natural products.

MNP	S	MW	QPLogP o/w	QPlogS	QPLogK	QPPCaco	QPlogBB	PHOA	RO5	JRO3	The most similar drug (%)
1	11	600.71	−3.39	2.00	−3.19	0	−5.32	0	3	1	Trientine (50)
2	1	640.76	1.53	−4.29	−0.83	0	−3.40	7	2	1	Sulfamazone (65.2)
3	11	818.97	2.00	−5.16	−0.84	0	−4.83	0	3	2	Dirithromycin (60.45)
4	11	798.96	1.78	−3.96	−0.91	0	−3.88	0	3	2	Monoxerutin (64.25)
5	1	638.74	1.00	−3.17	−1.01	0	−3.00	6	2	1	Sulfamazone (69.7)
6	5	718.85	2.16	−6.24	−0.52	0	−3.80	0	3	2	Azithromycin (65.70)
7	11	819.04	7.21	−10.30	1.40	36	−2.68	59	3	2	Rifaximin (56.25)
8	3	456.35	−1.04	−2.04	−1.47	0	−3.68	0	2	1	Methotrexate (79.50)
9	2	702.85	3.34	−4.86	−0.26	1	−3.12	9	3	2	Cefotiam (66)
10	1	599.60	3.22	−4.24	−0.13	9	−2.45	23	3	2	Amprenavir (68.5)
11	9	618.44	−2.24	−0.86	−1.25	0	−4.93	0	3	2	Lymecycline (67.23)

S (STARS) = Number of property/descriptor values falling outside the 95% range of similar values for known drugs. Recommended value 0–5. MW = Molecular weight Recommended values 130.0–725.0. QPlogPo/w = Predicted octanol/water partition coefficient. Recommended values –2.0–6.5. QPlogK = hsa Serum Protein Binding.Recommended values −1.5–1.5). QPlogS = Predicted aqueous solubility, log S. Recommended values –6.5–0.5. QPPCaco = Predicted apparent Caco-2 cell permeability in nm/sec. Recommended values < 25 poor, >500 great. QPlogBB = Predicted brain/blood partition coefficient. Recommended values –3.0–1.2. PHOA = Predicted human oral absorption on 0–100% scale. Recommended values > 80% is high <25% is poor. RO5 = Rule of Five, The rules are: mol_MW < 500, QPlogPo/w < 5, donorHB ≤5, and accptHB ≤10. Maximum is 4. JRO3 = Jorgensen Rule of 3 Violations. Maximum is 3.

Again, amongst the latter compounds, compound 10 showed a higher human oral absorption (23), as well as median aggregation to plasma proteins (Qlog k has serum protein binding: −0.13) and predicted aqueous solubility values (QPlog S: 3.22). Moreover, by considering the number of “stars” and the violations from the Lipsinki and Jorgensen rules, the obtained results for compound 10 indicated a high degree of reliability to be a drug candidate.

To find chemical similarity to the known drug molecules, the QikProp (Qikprop, 2015) software database identified five similar drug molecules for each entry according to its predicted descriptors. In this study, the results of two of the compounds were very promising. Accordingly, Azithromycin with a similarity of 65.70% was suggested for compound 6. It is interesting to know that in the early phase of COVID-19, azithromycin could reduce the need for hospitalization or duration of clinical recovery (Echeverría-Esnal et al., 2020; Million et al., 2020; Molina et al., 2020). There is also an opinion supporting the potential effectiveness of Azithromycin in SARS-CoV-2 infection, as well as its antiviral activity and immunomodulatory effects (Bleyzac et al., 2020). Interestingly, Amprenavir (Shen et al., 2010), as an antiretroviral protease inhibitor for HIV infection has been identified with 68.5% similarity to compound 10.

In conclusion, marine natural product 10, with high similarity to a known antiretroviral protease, might be considered as a potent inhibitor of TMPRSS2. However, molecular docking and MM-GBSA studies demonstrated that compounds 3, 4, 6, 7, 8, 9, and 11 were also able to inhibit TMPRSS2 as well. In a comparison study, these compounds showed better results than the standard TMPRSS2 inhibitors (camostat and nafamostat mesylate). Further computational, experimental, and clinical investigations are warranted to reveal their anti-SARS-CoV-2 activities.

## Data Availability

The original contributions presented in the study are included in the article/Supplementary Materials, further inquiries can be directed to the corresponding author.
