# Supplementary material for: Virtual Screening on Marine Natural Products for Discovering TMPRSS2 Inhibitors
Source: Front Chem. 2021 Oct 12;9:722633. doi: 10.3389/fchem.2021.722633 (PMC8545810; doi:10.3389/fchem.2021.722633)
Supplement: Supplementary file 1 [file DataSheet1.zip › Table 2.DOCX]

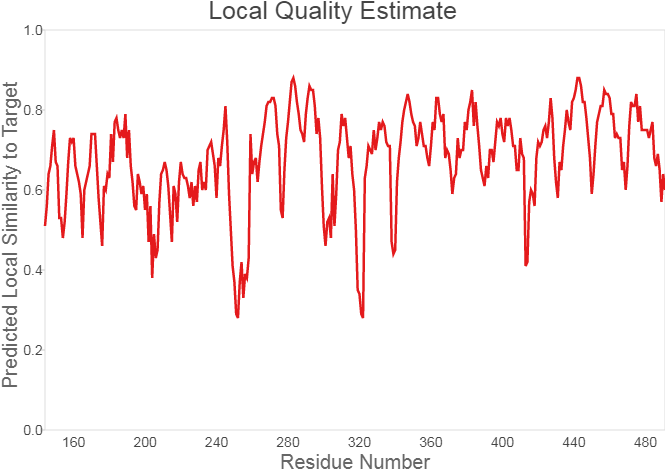


Figure S1. Local quality estimate of the derived model


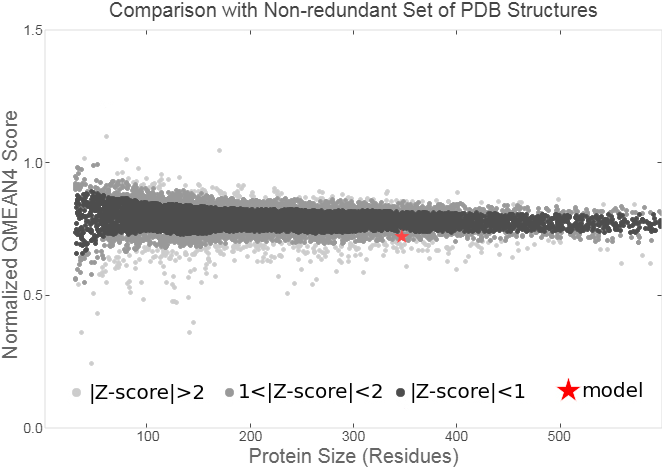


Figure S2. Normalized QMEAN4 score and its comparison with similar size of a set of PDB structures.
